# Surgeon’s experiences of receiving peer benchmarked feedback using patient-reported outcome measures: a qualitative study

**DOI:** 10.1186/1748-5908-9-84

**Published:** 2014-06-27

**Authors:** Maria B Boyce, John P Browne, Joanne Greenhalgh

**Affiliations:** 1 Department of Epidemiology and Public Health, University College Cork, Cork, Ireland; 2 Sociology and Social Policy, Faculty of Education, Social Sciences and Law, University of Leeds, Leeds, England

**Keywords:** Health status, Outcome assessment (health care), Patient reported outcome measures, Quality of life, Qualitative research, Quality improvements

## Abstract

**Background:**

The use of patient-reported outcome measures (PROMs) to provide healthcare professionals with peer benchmarked feedback is growing. However, there is little evidence on the opinions of professionals on the value of this information in practice. The purpose of this research is to explore surgeon’s experiences of receiving peer benchmarked PROMs feedback and to examine whether this information led to changes in their practice.

**Methods:**

This qualitative research employed a Framework approach. Semi-structured interviews were undertaken with surgeons who received peer benchmarked PROMs feedback. The participants included eleven consultant orthopaedic surgeons in the Republic of Ireland.

**Results:**

Five themes were identified: conceptual, methodological, practical, attitudinal, and impact. A typology was developed based on the attitudinal and impact themes from which three distinct groups emerged. ‘Advocates’ had positive attitudes towards PROMs and confirmed that the information promoted a self-reflective process. ‘Converts’ were uncertain about the value of PROMs, which reduced their inclination to use the data. ‘Sceptics’ had negative attitudes towards PROMs and claimed that the information had no impact on their behaviour. The conceptual, methodological and practical factors were linked to the typology.

**Conclusion:**

Surgeons had mixed opinions on the value of peer benchmarked PROMs data. Many appreciated the feedback as it reassured them that their practice was similar to their peers. However, PROMs information alone was considered insufficient to help identify opportunities for quality improvements. The reasons for the observed reluctance of participants to embrace PROMs can be categorised into conceptual, methodological, and practical factors. Policy makers and researchers need to increase professionals’ awareness of the numerous purposes and benefits of using PROMs, challenge the current methods to measure performance using PROMs, and reduce the burden of data collection and information dissemination on routine practice.

## Background

Patient reported outcome measures (PROMs) are questionnaires that assess patients’ views about their health [1,2]. They have traditionally been used to assess the burden of disease and to evaluate the clinical effectiveness of different treatments [2]. More recently, they have been used to give feedback to healthcare professionals in the hope that such information will lead to improvements in the delivery of care [3].

PROMs feedback can be based on data about individual patients or groups of patients defined at the level of the healthcare provider. Feedback about PROMs for individual patients is intended to help healthcare professionals identify new healthcare issues, assist in monitoring disease severity, and assess the effectiveness of current treatments [4-6]. For example, in an attempt to promote the effective management of chronic obstructive pulmonary disease symptoms patients were asked to complete the Short Form (36) Health Survey on a touch screen computer prior to their consultation, and feedback about the patient’s self-reported physical and mental health was provided to the physician during the consultation [7]. Feedback about PROMs for groups of patients seeks to stimulate professionals to consider their performance in comparison to their peers, empower purchasers and patients to select providers on the basis of performance, and facilitate reward mechanisms such as payment by performance [2,3]. For example, the NHS in England introduced the PROMs Programme in 2009 that mandated the collection of PROMs for patients undergoing four common elective procedures (hip replacement, knee replacement, hernia repair, and varicose vein surgery). Patients are invited to complete a disease-specific and a generic measure prior to and after their surgery. Patient data are aggregated to the level of the provider to compare performance and the results are publically reported online at NHS Trust level [2,8,9].

PROMs have been adopted as quality improvement tools in the UK [2,10], America [11,12], Australia [13-15], and Sweden [12]. In addition, Canada [16] and the Netherlands [17] have imminent plans to implement PROMs into healthcare policy. Arguably, the UK is revolutionising this field by firmly developing a role for PROMs in managing performance [18].

Despite the growing interest in PROMs, a number of systematic reviews have found weak evidence to support their effectiveness in promoting quality improvements [3,19-25]. A recent systematic review of 16 studies examined the impact on patient outcomes of feeding back PROMs data to healthcare professionals. The review found inconclusive evidence of the effectiveness of PROMs feedback about individual patients. Only one study examined the effectiveness of peer benchmarking using PROMs data. This study found no statistically significant difference in patient outcomes between the feedback and control arms [3]. In addition, a recent review of the qualitative literature found 14 studies that had explored professional’s views on the value of receiving PROMs feedback about individual patients [26]. A further two studies had examined the value of PROMs feedback at both the individual and aggregated level, but it was not possible to separate the results for these different forms of feedback [26]. Given that the use of PROMs at the aggregate level presents potentially unique challenges, it is important to examine professional’s views and experiences about this specific form of feedback [27]. For example, aggregated PROMs data may prove more difficult to interpret than PROMs data about individual patients, and peer benchmarking may be mistrusted because the methods used to perform case-mix adjustment of PROMs data are not widely understood [27,28]. These issues may engender confusion and scepticism among those tasked with using the data for quality improvement purposes [29].

This study explores professional’s experiences of using PROMs as peer benchmarking tools. The objectives of this research were to identify the practical challenges of collecting and using PROMs data in practice, methodological challenges associated with generating useful PROMs feedback, attitudes towards the value of this feedback, and the impact of this information on stimulating changes to clinical practice and on promoting professionals to undertake additional audit or research activities. This research is timely considering the current plans to expand the NHS PROMs Programme to different conditions and to begin publishing data at the individual consultant level [2,18].

## Methods

### Design overview

This paper reports on a qualitative research study that was nested within a larger randomised controlled trial of PROMs feedback. The trial was titled the Patient Reported Outcome: Feedback Interpretation and Learning Experiment (PROFILE) trial—refer to (ISRCTN 69032522) for more details. PROFILE trial aimed to evaluate the effectiveness of the NHS PROMs Programme methodology for surgeon level feedback in an Irish context [1]. In brief, PROFILE tests the hypothesis that healthcare professionals who receive benchmarked PROMs feedback will have better future outcomes than those who do not receive feedback. This trial was undertaken in Ireland where performance monitoring has not yet progressed beyond measuring processes such as waiting times, length of stay, and adherence to hygiene standards. This was the first time the participating surgeons had received peer-benchmarked feedback about their patient outcomes. We discuss the methodology of the trial below and describe the nature of the PROMs feedback provided to clinicians within the trial. We then subsequently explain the methodology of the nested qualitative study.

PROFILE is a trial of 21 high-volume hip replacement surgeons and their patients. In the trial, patients were asked to fill out a questionnaire before and six months after their operation. Questionnaires included demographic questions on the patient’s age, gender, duration of symptoms, and the PROMs included were the Oxford Hip Score (OHS) [30], the EQ-5D [31], a shortened version of the Hip Osteoarthritis and Outcome Score (HOOS) [32], and a general health status item. Post-operative questionnaires were similar except they also included questions on the results of the operation and post-operative problems, including allergy or reaction to a drug, urinary problems, bleeding, and wound problems [1]. Pre-operative data collection took place in a pre-assessment clinic, if available, or alternatively when the patient was admitted to the hospital for surgery. The data collectors included nurses and registrars. Post-operative data collection was managed by the research team using a postal survey. Questionnaires were posted to patients six-months after their surgery, and a reminder was sent four weeks later if a reply was not received within this timeframe.

**Figure 1:**
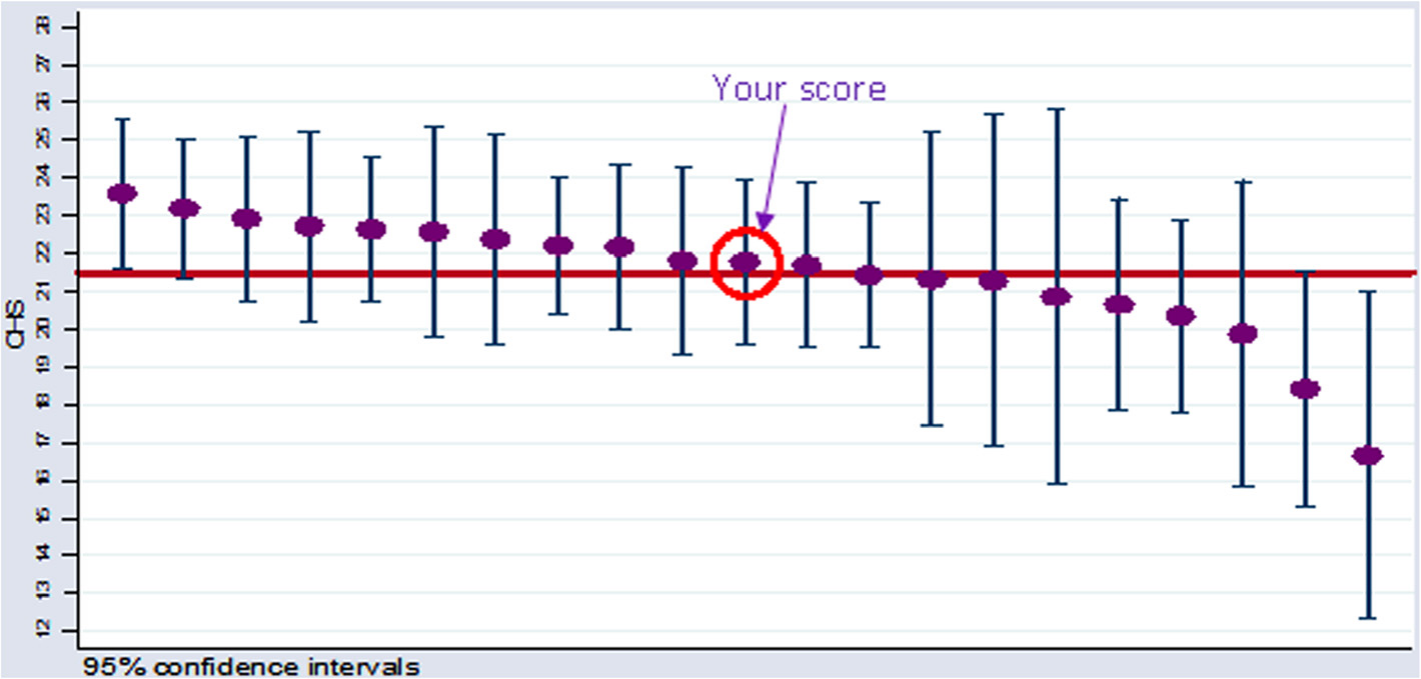
An example of peer benchmarked PROMs feedback.

The data collection occurred in two phases: pre- and post-feedback. The pre-feedback phase was used to generate peer benchmarked PROMs reports for the 11 surgeons randomised to the intervention arm of the trial. The content of the feedback report was based on research which examined clinician’s preferences on metrics used to compare surgical performance [33] and included the mean change (post-operative minus pre-operative) in the OHS, the proportion of patients that reported improvements in their hip problem, and the proportion of patients that reported having at least one of four problems after surgery. Case-mix adjustment of the OHS was used to ensure a fair comparison of surgeon level results. The OHS was adjusted to account for patients’ pre-operative OHS, age, gender, general health status, and mental health status. Surgeon’s scores were clearly highlighted for each outcome demonstrating how they performed in comparison to the other 20 surgeons in the trial; however the identity of these surgeons remained anonymous (Figure 1). The feedback report was based on data from 759 patients. A minimum patient recruitment was set at 32 patients per surgeon—a requirement that was necessary to accurately benchmark outcomes. The post-feedback phase of the trial follows the same data collection procedures on a new cohort of patients. In this phase, PROMs act as the outcome measure by examining differences between the feedback and control arms. Follow-up data collection for PROFILE is currently ongoing, and the results will be published in late 2014. Feedback was provided in January 2013 and the interviews were performed between three and five months later.

### The qualitative study

This paper reports on a qualitative study that was nested within the PROFILE trial described above. The qualitative study employed a Framework approach [34]. This is appropriate when aiming to generate policy-orientated findings and recommendations for practice in a field where an existing conceptual framework derived from the literature was an appropriate starting point for the data collection and analysis [35,36].

### Sampling and data collection

All 11 surgeons in the feedback arm of the PROFILE trial were invited to participate in a face-to-face interview, and consented to do so. Given that this represents a complete capture of all possible respondents of interest, the sampling method can be characterised as a census. The participants varied in terms of the setting of their usual workplace, their relative performance ranking and their previous experience of using PROMs (Table 1). The 10 surgeons in the control arm were not interviewed because they did not receive feedback, so their reactions to this information could not be elicited.

**Table 1:** Characteristics of participants

**Sex**	**Setting**	**Above/below average (OHS)**	**Experience of using PROMs**
Male	Public	Above	None
Male	Public	Above	Minimal
Male	Public	Below	Moderate
Male	Public	Below	Minimal
Male	Public	Below	Minimal
Male	Private	Above	Minimal
Male	Mixed	Above	Moderate
Male	Mixed	Above	Minimal
Male	Mixed	Above	None
Male	Mixed	Below	None
Male	Mixed	Below	Minimal

The topic guide was informed by the objectives of the research and the results of a systematic review undertaken by the authors to synthesize existing qualitative evidence on professional’s experiences of using PROMs as quality improvement tools [26]. This review identified four themes: practical considerations, attitudes towards PROMs, methodological concerns, and the impact of the feedback on care. A draft discussion guide was developed from these themes. This was reviewed by the research team and independently with clinical professionals before finalising the discussion points. The final guide covered five topics: experiences of using PROMs, attitudes towards using PROMs as peer benchmarking tools, methodological factors, practical factors with collecting and using PROMs data, and the impact of the information on behaviour (see Additional file 1).

The interviews were performed by MB, who is a trained health services researcher with seven years’ experience working in both qualitative and quantitative approaches that reflects a pragmatic paradigm underlying this research. Before commencing each interview, the rationale for the study and the specific purpose of the discussion was clearly outlined to participants. Each surgeon provided written consent for digital recording and verbatim transcription. The study was conducted according to ethical guidelines [37]. The Research Ethics Committee of the Cork Teaching Hospitals (CREC) approved the study protocol, as well as the ethics committees within the hospitals.

### Data analysis

A Framework approach was employed to analyse the data [34]. Framework analysis uses a stepwise approach to ensure a systematic, rigorous, and transparent approach to the analysis [36]. QSR International’s NVivo 10 software was used to assist with the analysis [38]. First, the raw data were repeatedly read to identify initial concepts. A ‘one sheet of paper’ mapping exercise [39] developed these ideas into a preliminary framework. This framework was tested by labelling (indexing) a sample of the data, and was revised before being populated by the entire dataset. Next, the data were categorised and synthesised by sorting and summarising the material into charts. The raw data were exported into these charts to ensure the meaning and context of the participants views were retained. Lastly, patterns within the data were examined to help describe and explain the findings by sequentially comparing each theme against the other four themes and across different cases [34]. The typology emerged from two themes, and differences between the remaining three themes were examined against the typology. A framework was developed to describe the relationship between the themes by examining subtle differences across the three types of participants. The characteristics of the participants were examined in a similar manner to produce explanations for the groupings.

An academic clinician independently coded three randomly selected transcripts and helped develop and refine the framework prior to commencing the indexing. As the authors are not clinicians, this perspective ensured the analytic framework evolved with a sensitivity to the culture of the Irish healthcare system. JB and JG participated in discussions about the analytic framework throughout the process. Regular analysis meetings between the authors challenged the analytic process, interpretation of the data, and any possible observer bias. MB kept a reflective journal during the analysis and used personal memos to track decisions and challenge any personal or professional biases in interpreting the data.

Given the elite position of surgeons, it has been noted that relatively few participants (between six and twelve) may offer deep insights [40,41]. Therefore, the framework was developed after eight interviews were completed. The final three interviews were used to examine saturation. This was undertaken by comparing the themes emerging from each additional interview against the framework to establish if any new issues or concepts emerged [42].

### Rigour

We took a number of steps to enhance the trustworthiness of the study finding [43]. First, we examined previous research to frame the findings. Second, we built trust with the study participants by clearly explaining the research aims, declaring the researcher’s independent affiliation and assuring the interviewees that their confidentiality would be maintained. Third, we sought peer scrutiny throughout the study by involving healthcare professionals when drafting the discussion guide, checking shared meaning of concepts by jointly coding transcripts with an independent clinician, and sharing ideas with the research team throughout the development of the framework and when defining themes. Fourth, the lead interviewer maintained a reflective approach throughout the research by writing a journal during the data collection phase and keeping memos throughout the analysis phase. Transferability was enhanced by recruiting participants from 16 organisations across mixed settings and with mixed levels of experience, and by providing rich information on the study context and findings to enable future researchers to draw comparisons. Dependability was enhanced by clearly describing our methods to enable study replication. Confirmability was promoted by recognising study limitations and by declaring the researcher’s beliefs and assumptions [43].

## Results

All 11 consultants in the feedback arm of the trial agreed to participate. All participants were male, six worked in a public setting, five in both public and private settings, and one in a private setting only. Six surgeons had above average OHS scores and five had below average OHS scores when all surgeons were benchmarked against each other. Two had moderate experience, six had minimal experience and three had no experience of previously using PROMs (Table 1). Interviews were held privately in the participant’s workplace. The mean length of the interviews was 42 minutes (range 15 to 84); the longer interviews tended to focus more thoroughly on the methods.

Five themes were initially identified: conceptual (understanding PROMs), methodological (focus, accuracy and interpretation of the data), practical (issues with collecting and using the data), attitudinal (valuing the information), and impact (using the information to make changes to the processes of care). Subsequently the themes about ‘attitudes’ and ‘impact’ were merged due to their co-dependency on participant’s reactions to the feedback. Quotations were selected to represent the essence of each sub-theme and have been coded to protect the subject’s confidentiality (Table 2).

**Table 2:** Themes, sub-themes and excerpts from the participants

**Themes**	**Sub-themes**	**Excerpts**
Conceptual	Subjective measurement	‘Getting patients to fill out forms is grossly inaccurate in my book…the patient 9 time out of 10 wouldn’t understand what hip pain is’ (S9)
		‘There is some subjective element but it is a reasonably validated objective assessment’ (S2). ‘Well they are partly objectified, aren’t they?’ (S11)
		‘I suppose the difference maybe with my results is the difference between the maybe more objective measures and the subjective measures’ (S5)
	PROMs V Satisfaction	‘Patient satisfaction in a sense is a balance between what their expectations were beforehand and what they achieved afterwards’ (S10)
		‘You know there is one outcome there on how much the patient likes the outcome as I like to call it’ (S2)
		‘When they are not perfect, they manifest that by saying they are quite poor’ (S7)
	PROMs V clinical data	‘Clinically I see very very very few problems and very few dissatisfied patients…that is just wrong. I am sorry I just can’t accept that’ (S10)
Methodological	Focus and variability	‘You should concentrate on operations that have dubious results’ (S8)
		‘The increments between each surgeon are tiny …I mean your spread there between top and bottom is only six points’ (S7)
	Timing	‘To see if there was any differences at four to six weeks’ (S4)
		‘The other thing is the timing is critical because one would generally not measure anything in hip surgery and knee surgery for at least one year’ (S11)
	Choice of measures	‘That score has issues with validity for certain age groups’ (S1)
		‘The patient might perceive it as a complication but it is not, it is part of the normal process’ (S8)
		‘You know it has to be patients with a problem after surgery that is directly related with the surgery’ (S10)
	Interpretation	‘Unless I was able to compare myself against somebody else who does things quite differently’ (S2)
		‘I mean strictly speaking someone that is at the tail end should be at the tail end in all three’ (S7)
	Validity (data quality, case-mix adjustment, sampling)	‘Something is wrong somewhere: either they have problems and they are not telling me or else there is something odd in data collection’ (S10)
		‘Even if you adjust them it is not going to give you the proper information’ (S1)
Practical	Time	‘If I had time, maybe. I don’t have time. I mean, I have continuous ideas…and am…let’s say resolutions to measure outcomes better and more often and all the rest of it but we don’t have the time like and we don’t have the staff’ (S11)
	Support	‘No interest. No support. No help. No funding’ (S2)
		‘We don’t have anything strictly audit related because the big problem with the hospital audits is the information gathering is poor’ (S7)
		‘You need generally a political will to get it because it can achieve nothing but to cost them more’ (S2)
		‘You need software, you need somebody to analyse it’ (S3) ‘…that takes help, statistical help’ (S4)
Attitudinal	Value	‘There have been a lot of high profile problems in recent times and maybe these kind of problems would have been spotted sooner if we were collecting this type of data’ (S5)
		‘You see your patients and they are happy but in general terms you don’t know how you are performing compared to your peers’ (S4)
	Undecided	‘That is kind of a relatively disappointing figure, I would have thought and not just mine, I think the overall is kind of a little bit disappointing. Why it is? I am not sure’ (S3)
	No value	‘I just think there is a lot of effort being put in there for not a lot of surgical gain from my perspective’ (S8)
Impact	Impact	‘I am going to try and do it better’ (S4)
		‘I went off for a few days and started thinking about things so even though my results would appear not to be brilliant, it was very beneficial for me’ (S7)
	No impact	‘I seem to be in the middle there and I wouldn’t be changing what I do on the basis of it’(S2)
		‘Unfortunately, it does not provide me with one iota that helps me make my next score any better’ (S10)

### Theme one: conceptual—understanding PROMs

Participants varied in their understanding of PROMs as a concept. This became evident in three ways: comprehending subjective measurement, confusing PROMs with patient satisfaction measures, and aligning PROMs with clinical data.

### Subjective measurement

Participants declared a respect for eliciting information from patients, but expressed concern about the scientific properties of PROMs. There was an underlying doubt about patient’s ability to report on issues such as pain and physical function. Surgeons consciously deliberated the concepts of subjectivity and objectivity. PROMs data were seen by many as ‘subjective’ and therefore less trustworthy. However, the distinction was not absolute as they ranked different PROMs by their level of ‘objectivity’.

### PROMs versus satisfaction

Consultants often did not distinguish the difference between PROMs and measures of patient satisfaction or experience, and thus assumed that the questionnaires captured information on the processes of care throughout their healthcare journey.

### PROMs versus clinical data

Participants expected PROMs data to align closely with clinical indicators. Many expressed disbelief about the percentage of patients who reported that they had not improved or had a problem after surgery. Surgeons felt that these figures did not match their experience of clinical practice and verbal feedback from patients post-operatively.

### Theme two: methodological—measurement decisions, measurement accuracy and interpretation

This theme captured methodological issues around the focus of measurement, the timing of data collection, the choice of measures, trust in the accuracy of the PROMs feedback, and problems with data interpretation. A key underlying issue within this theme were the methodological threats to the trustworthiness of PROMs as an indicator of surgeon’s performance.

### Focus of measurement

Participants questioned the rationale for focusing on hip replacement surgery. One consultant queried the cost-effectiveness of concentrating on a procedure where poor outcomes are perceived to be rare. Some surgeons discussed the relatively small variability between surgeons and, therefore, the clinical value of performance management in a field where only marginal improvements may be possible at the population level.

### Timing

Participants discussed the timing of the post-operative data collection. One participant was interested in the rate at which patients recover from different approaches and techniques, and how this would influence performance ranking at different time points, particularly in the short term. Others believed that six-month follow-up was too soon because patients continue to improve for up to a year.

### Choice of measures

Participants also recognised that the measures collected influenced the value of feedback. One surgeon questioned the appropriateness of the OHS because it was developed for an older population with arthritic problems. The choice of measures became particularly pertinent when participants considered the data about post-operative complications. Some felt that it was unfair to associate these complications with their performance because they believed that the specific problems in question were not a direct complication of surgery.

### Accuracy of the feedback

Interviewees expressed a range of opinions about the validity of PROMs. The factors identified were related to possible biases, confounding, and chance.

Participants were aware that incorrect administration and completion of the measures would affect the data quality. In particular, they were concerned about the potential to manipulate scores by failing to recruit patients who may be more likely to have a poor outcome, thus creating a selection bias. Incorrect completion of the measures was identified as a possible source of information and recall bias. Participants questioned the patient’s ability to complete the PROMs correctly. This was considered especially relevant for patients with co-morbidities who might confuse problems arising from their hip osteoarthritis with problems arising from other conditions. Concern was also expressed about the possibility that patients with low literacy might tick random answers or ask family/friends to complete the questionnaire on their behalf. Participants were also worried about the influence of patient expectations on PROMs, which might lead to an underestimation of the ‘true’ outcome. Others argued that patients might deliberately underestimate their pre-operative outcomes in the belief that the information was being used to ration care. However, one participant identified a scenario where patients may overestimate their outcome due to a ‘post-event rationalisation’ , where patients start to justify their choice to have the operation, resulting in a belief that their outcome is better than it actually is.

The issue of confounding was identified as a serious threat to the accuracy of the findings. Consultants were concerned about the impact that patient case-mix, differences in resources across hospitals and differences in support services at a community level had on patient outcomes. Patient level confounding was perceived as the most serious threat and many were sceptical about the accuracy of adjusting for case-mix.

Lastly, some surgeons were concerned about the influence of chance on findings. Surgeons were interested to see if their ranking would be similar with a larger sample or different samples of patients. Therefore, many were keen to receive additional feedback reports to monitor their performance.

### Interpretation of the feedback

Consultants had difficulty making sense of the PROMs feedback. Understanding the variation between and within surgeons was challenging. Surgeons also found it hard to identify opportunities for quality improvement within the feedback.

Consultants had problems identifying reasons for variation between surgeons because of the number of causal factors linked to PROMs. Participants found that the PROMs feedback alone was insufficient to provide explanations for poor performance. However, some thought that linking PROMs to information about clinical practices might improve future decision making. Finally, some aspects of the feedback confused certain participants who ranked differently across the outcome measures because they could not explain the reasons for such deviations.

### Theme three: practical issues with collecting and using the data

The process of collecting and using PROMs data created barriers to a positive engagement with the exercise. Data collection added to workload pressures. Many surgeons stated that their support staff were not willing to accept the increased workload associated with questionnaire administration. Furthermore, surgeons recognised that political will at a hospital and system level was necessary to maintain such initiatives because real quality improvements often require a level of resource flexibility. In addition, there was concern that both clinical and managerial professionals lack the knowledge and training to use PROMs data. Surgeons recognised that in the absence of such training there was a danger that the data may be inappropriately used.

### Typology: attitudes (valuing the data) and impact (using the data)

Three distinct groups emerged with respect to views about the final themes: attitudes (the value attached to PROMs) and impact (the likelihood of using PROMs to change clinical practices). Two surgeons (Advocates) expressed a positive attitude to the feedback they received and stated that the information had an impact by promoting a reflective process focusing on their clinical practice, although they did not explicitly state specific changes to the process of care. One of these surgeons stated that the results provided additional motivation to continuously aim to perfect his technique. The other stated that the results promoted a process whereby he considered at depth the aspects of care that may have affected performance.

A separate group of four surgeons (Converts) were uncertain about the value of PROMs, and this reduced their inclination to use the data. They lacked the knowledge to make an informed decision on the usefulness of PROMs but were reassured that their performance was similar to their peers. This group generally felt that it is important to know what patients think about their outcome but emphasised the need to provide actionable feedback to professionals.

A third group of five surgeons (Sceptics) believed that the PROMs feedback they had received was not clinically useful and so the feedback had no impact on their behaviour. They felt that there were too many scientific concerns to trust the data, that the data collection was cost-ineffective, and that the data were not a useful source of ideas about ways to stimulate improvement.

### Relationship between themes—a conceptual model

**Figure 2:**
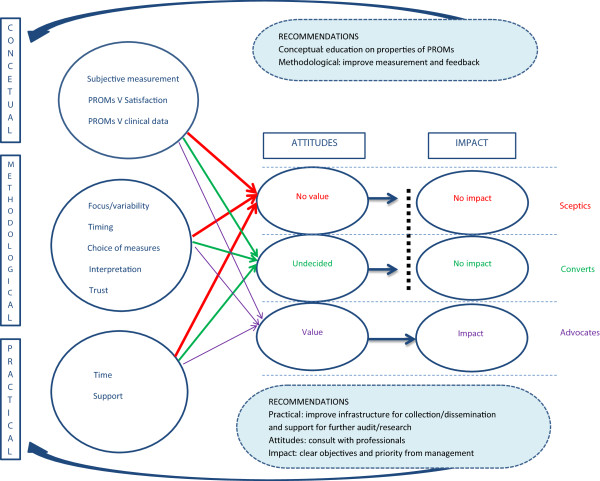
A conceptual framework of the relationship between themes.

A matrix helped examine patterns in the themes (Table 3). By examining the patterns between the themes and the typology, it became clear that the conceptual, methodological, and practical issues were important determinants of professional’s attitudes towards PROMs. The attitudes, in turn, defined the impact of the information on behaviour. A conceptual framework was developed to depict the relationship between the themes (Figure 2).

**Table 3:** Mapping of themes and sub-themes across surgeons

	**Characteristics**	**Typology**	**Conceptual**	**Methodological**	**Practical**
**Surgeon 4**	**Baseline performance**	Advocate	PROMs V Satisfaction	Interpretation	Support/infrastructure
	**(OHS):** Above average	(value and impact)		Timing	
	**Setting:** Mixed			Validity	
	**Experience:** Moderate				
**Surgeon 7**	**Baseline performance**	Advocate	PROMs V Clinical	Interpretation	Support/infrastructure
	**(OHS):** Below average	(value and impact)		Focus/variability	
	**Setting:** Public			Validity	
	**Experience:** Minimal				
**Surgeon 2**	**Baseline performance**	Convert	Subjective measurement	Interpretation	Time/workload
	**(OHS):** Below average	(undecided and no impact)	PROMs V Satisfaction	Validity	Support/infrastructure
	**Setting:** Public				
	**Experience:** Moderate				
**Surgeon 3**	**Baseline performance**	Convert	PROMs V Satisfaction	Interpretation	Time/workload
	**(OHS):** Above average	(undecided and no impact)	PROMs V Clinical	Focus/variability	Support/infrastructure
	**Setting:** Private			Validity	
	**Experience:** Minimal				
**Surgeon 5**	**Baseline performance**	Convert	Subjective measurement	Interpretation	Time/workload
	**(OHS):** Above average	(undecided and no impact)	PROMs V Satisfaction	Focus/variability	Support/infrastructure
	**Setting:** Public			Timing	
	**Experience:** None			Validity	
**Surgeon 6**	**Baseline performance**	Convert	Subjective measurement	Interpretation	Support/infrastructure
	**(OHS):** Below average	(undecided and no impact)		Validity	
	**Setting:** Public				
	**Experience:** Minimal				
**Surgeon 1**	**Baseline performance**	Sceptic	n/a	Interpretation	Time/workload
	**(OHS):** Above average	(no value and no impact)		Measurement	Support/infrastructure
	**Setting:** Public			Timing	
	**Experience:** Minimal			Validity	
**Surgeon 8**	**Baseline performance**	Sceptic	Subjective measurement	Interpretation	Time/workload
	**(OHS):** Below average	(no value and no impact)	PROMs V Satisfaction	Focus/variability	
	**Setting:** Mixed			Validity	
	**Experience:** None				
**Surgeon 9**	**Baseline performance**	Sceptic	Subjective measurement	Interpretation	n/a
	**(OHS):** Above average	(no value and no impact)		Focus/variability	
	**Setting:** Mixed			Timing	
	**Experience:** None			Validity	
**Surgeon 10**	**Baseline performance**	Sceptic	Subjective measurement	Interpretation	Support/infrastructure
	**(OHS):** Below average	(no value and no impact)	PROMs V Satisfaction	Focus/variability	
	**Setting:** Mixed		PROMs V Clinical	Timing	
	**Experience:** Minimal			Validity	
**Surgeon 11**	**Baseline performance**	Sceptic	Subjective measurement	Interpretation	Time/workload
	**(OHS):** Above average	(no value and no impact)	PROMs V Satisfaction	Focus/variability	
	**Setting:** Mixed		PROMs V Clinical	Timing	
	**Experience:** Minimal			Validity	

There was evidence that surgeons’ understanding of PROMs was an important determinant of the extent to which they might value and use the data. The ‘Converts’ and ‘Sceptics’ were more likely to deliberate the distinction between subjective and objective measurement, placing more trust in the scores that were perceived to be more ‘objective’ , and were more likely to misinterpret the information.

The strongest influence on surgeon attitudes and behaviour was the methodological theme. The ‘Advocates’ focused less on the factors that may impact on the data quality and more on further research opportunities to investigate the reasons for variations in outcomes, such as examining the relationship between outcomes and expectations, exploring rankings at different time periods, and undertaking case-study reviews. The ‘Converts’ tended to appreciate aspects of the feedback but were perturbed by some of the methodological issues. Their discussion focused in more detail on the possible errors in the data, particularly the impact of incorrect administration and completion of the questionnaire on data quality. Similarly, these professionals highlighted inconsistencies between the PROMs scores deliberating whether the divergences were associated with inaccuracies in the data. The ‘Sceptics’ focused on reasons why they did not trust the data. They also highlighted the impact of incorrect completion and administration on findings, and questioned the measurement properties of PROMs, the focus on hip replacement surgery, and the complexity of causal factors determining outcomes.

The views of the groups also differed with respect to their concerns about practical issues. The ‘Advocates’ focused on how PROMs could be used more effectively if there was greater audit and research support. The ‘Converts’ focused on the impact on workload, the lack of collaboration between staff and management, and the cost of data collection. The ‘Sceptics’ provided an insight into the negative consequences of collecting PROMs, including the opportunity costs involved, and were cynical about the willingness and ability of their local hospital to support real quality improvements.

There was no obvious relationship between surgeon responses and their performance ranking or the setting in which they worked. However, previous experience with using PROMs may have influenced their responses. Two surgeons had experience of collecting PROMs routinely in practice: one of these was classified as an ‘Advocate’ and one as a ‘Convert’. Five surgeons had minimal experience of using PROMs for research purposes: one was classified as an ‘Advocate’ , two as ‘Converts’ and two as ‘Sceptics’. Three surgeons claimed they had no experience with using PROMs: one was classified as a ‘Convert’ and two as ‘Sceptics’.

## Discussion

This is the first study of healthcare professionals’ experiences of receiving peer-benchmarked feedback using PROMs. Three groups of surgeons emerged from the analysis: Advocates, Converts, and Sceptics. ‘Advocates’ had positive attitudes towards the use of PROMs and admitted that the information had an impact on their behaviour by promoting a reflective process on their clinical practice. ‘Converts’ had mixed attitudes because they were uncertain about the value of PROMs, which prevented them from using the data to inform their practice. ‘Sceptics’ portrayed negative attitudes towards the value of PROMs and reported that the feedback had no impact on their behaviour. The barriers towards the use of PROMs information may be categorised into conceptual, methodological and practical factors.

Conceptual issues refer to problems with understanding PROMs, for example, comprehending subjective measurement, confusing PROMs with patient satisfaction measures, and aligning PROMs with clinical data. These problems were more common among the ‘Converts’ and ‘Sceptics,’ which may be partly linked to an unfamiliarly with using these measures. Though based upon a small sample size, this is tentative evidence that familiarity with PROMs is associated with a more positive disposition towards their use. Methodological concerns, for example, the focus of measurement, the timing of data collection, the choice of measures, the validity of the information, and interpretation of the data were further barriers to full engagement with PROMs. The ‘Advocates’ used the information to prompt ideas for further investigations. In contrast, ‘Converts’ and ‘Sceptics’ were more likely to question the data quality and less likely to accept responsibility to further explore the reasons for variations in performance. Finally, practical constraints such as workload pressures and a lack of support were also barriers towards the uptake of PROMs. Practical issues were more of a concern for the ‘Converts’ and ‘Sceptics’. This may be because the ‘Advocates’ already had some of these processes in place. However, implementing the routine use of PROMs not only requires dedicated staff time for data collection but also appropriate information technologies, statistical support, and resource flexibility to appropriately use the information, which can be difficult to procure.

### Implications of findings

These findings outline the barriers to the effective implementation and use of PROMs in practice. The conceptual framework produced by this research can be used by practitioners, managers, and policy makers who hope to use PROMs benchmarking to improve the quality of care and by researchers who are interested in the implementation of these strategies.

Some participants were familiar with using PROMs for research projects or had experience collecting PROMs in practice to manage patient care; however the use of PROMs as performance measures was a new concept for most of the surgeons. This inexperience may have led them to make sense of PROMs by relating or equating them to measures they were familiar with in a performance monitoring context, such as clinical indicators like revision rates and patient satisfaction surveys. However, these were not measured in this study. PROMs address unique constructs and perform a unique role in health measurement [44-49]. These findings highlight that providing training on the different functions of PROMs, the measurement properties of the instruments and the interpretation of the data is necessary if PROMs are to be effectively used in practice. Furthermore, co-designing feedback reports with professionals would generate information that professionals perceive as useful and increase the likelihood of positive engagement [33,50]. Further qualitative research could be used to assess whether opinions of surgeons change as they receive PROMs feedback and become more familiar with the data.

The research highlights many interesting methodological questions for future research studies. The recent application of PROMs as performance monitoring tools creates uncertainty regarding the adequacy of the existing measures. Many of the tools were developed to assess the effectiveness of healthcare interventions across patient populations, but have been subsequently applied in clinical practice for individual patient-level evaluations and to detect differences in quality of care between healthcare professionals [27]. This creates problems as the reliability and validity of the information generated at these levels cannot be guaranteed [51,52]. Another issue to consider is that PROMs data are not directly ‘actionable’ in that they do not point to solutions that will improve the quality of care. PROMs produce scale-level data that summarise the responses to a number of items. Scale-level data are less meaningful than item-level data from a clinical perspective and is more suited to establishing ‘that’ differences exist, as opposed to ‘why’ they exist. A possible solution to these measurement and interpretation issues is to adopt psychometric techniques such as Rasch modelling. Rasch analysis has the capability of producing more precise measurement instruments and enables the interpretation of the information at the item and scale level [53,54].

Surgeons identified the need to produce meaningful and useful feedback, suggesting that PROMs data should be provided alongside clinical and patient experience data because this may offer an insight into the factors causing variation. Our knowledge about how these perspectives correlate across the range of measures is not well advanced. For example, a review examining the relationship between satisfaction with care and PROMs found a positive correlation, however the causative direction of this relationship could not be determined [55]. The evidence on the relationship between improving processes of care and outcomes is also weak [56]. This may be a symptom of inadequate efforts to generate high data quality and to test the use of these measures in practice prior to their routine introduction. However, it is important to recognise that ongoing developments in both process and outcome measures and measurements are necessary to drive a deeper conceptual understanding of the link between these elements of care [56,57].

The focus of measurement also needs to be considered, as performance monitoring will have the greatest impact when the variation between professionals is large or baseline performance is poor. Hip replacement may not be the most sensible procedure to target, as this study found that the variation between surgeons was small and baseline performance was good [58].

The wider outcomes literature has identified some additional attributes of successful performance improvement initiatives [59,60]. There is evidence that a meticulous focus on generating high-quality data can promote positive changes in outcomes over time, particularly for ‘bad outliers’ [59], and that collaborative improvement programmes can stimulate improvements far more quickly than efforts by single providers [60]. The benefit of a collaborative programme is that large sample sizes enable a robust assessment of relationships between process and outcomes, identifying best practices that can be rapidly rolled out to the entire group. This in combination with an increased focus on creating an appropriate environment for quality improvement can lead to better patient outcomes [60]. Our study similarly highlights that building for a momentum for change depends on effective leadership and ongoing practical support to help professionals identify where improvements are required [3].

### Study limitations

There are some limitations to this research. First, the research is based on the views of only eleven participants. However, it should be acknowledged that consultants are an ‘elite’ source of insight, given their authority and in-depth knowledge of the system [41]. In addition, established methods were used to assess if data saturation was reached [42]. Nevertheless, the generalisability of the findings to other types of healthcare professionals should be considered. Second, the impact of performance measurement is dependent on various contextual factors such as local culture and governance structures. This research was undertaken in Ireland, where professional performance assessment is still at a rudimentary level; therefore professionals may have had a general suspicion of peer benchmarking. Third, the research is based on only one round of feedback. Professionals may be more likely to engage with PROMs data if they receive regular feedback reports and can observe meaningful trends over time. Fourth, qualitative research will not capture the psychological impact of measurement on behaviour such as the Hawthorne effect, which may lead to more subtle changes to practice. Finally, this research does not explore the influence of feedback on the wider healthcare system. The NHS PROMs programme provides feedback at the NHS Trust level that engages different aspects of the clinical governance infrastructure and may provide useful information to different actors such as patients and purchasers.

## Conclusion

Interest in the use of PROMs as quality improvement tools is growing. However, this research demonstrates that there are conceptual, practical, and methodological issues that determine attitudes towards the use of PROMs and, in turn, professionals’ willingness to use the information to inform practice. Policy makers and researchers need to engage more effectively with professionals, provide sufficient education and training, develop better measures and feedback mechanisms, and help to build a more supportive and efficient data collection infrastructure.

## Abbreviations

PROMs: Patient reported outcome measures; NHS: National Health Service; PROFILE: Patient Reported Outcomes: Feedback Interpretation and Learning Experiment; OHS: Oxford Hip Score.

## Competing interests

The authors declare that they have no competing interests.

## Authors’ contributions

MB was involved in the conception, design, analysis and interpretation of data. JB was involved in the conception, design, analysis and interpretation of data. JG was involved in the design, analysis and interpretation of data. All authors were involved in drafting the article and revising it critically for important intellectual content, and approved the final version to be published. MB is the guarantor.

## Supplementary Material

Additional file 1 Discussion guide for interviews. Click here for file
