# Supplementary material for: Surgeon’s experiences of receiving peer benchmarked feedback using patient-reported outcome measures: a qualitative study
Source: Implement Sci. 2014 Jun 27;9:84. doi: 10.1186/1748-5908-9-84 (PMC4227108; doi:10.1186/1748-5908-9-84)
Supplement: Additional file 1 — Discussion guide for interviews. [file 1748-5908-9-84-S1.docx]

**Appendix 1- Discussion guide for interviews**

*Firstly, I would like to thank you for taking the time to talk to me. My name is Maria Boyce, I am a researcher based in UCC. I would like to explain the background behind this research and rationale for the interview. I will then ask you to sign the consent form before we commence the interview.*

*I am going to use the term PROMs throughout the interview. This stands for Patient-reported outcome measures which are questionnaires that assess patients’ health including: symptoms, function, well-being, health-related quality of life (HRQOL)* and other health-related constructs.

As you may know, the NHS introduced a national programme in 2009 which made the collection of PROMs a mandatory requirement for audit. Therefore, every patient that receives a hip or knee replacement surgery is asked to complete a questionnaire to assess their pain and function before and six months after their operation. The results are compared at a hospital level and are publically reported online to inform patient choice. They intend to extend the use of PROMs to other areas such as mental health, oncology and some chronic conditions, and they have also plans to link payments to results. This programme is stimulating much interest from policy makers internationally. However, there is little empirical evidence on the use of PROMs as a performance measure and so this study is the first to evaluate the usefulness of this strategy. Furthermore, professionals are the target of such an initiative but there has being no attempt to evaluate their views on the usefulness of such data and therefore this is the focus of this interview.

I am undertaking an interview with every surgeon in the feedback arm of the PROFILE trial. You received benchmarked feedback after Christmas which was based on PROMs data. I would like to establish your views on the collection and value of such data. The interview should last about 30-40 minutes. I would just like to check a few things before we get started.

- Would you mind if I record this interview? Anything we discuss will be confidential and your identity will remain anonymous on any reports or publications. Finally you can stop the interview at any point, if you wish. Do you have any questions for me before we get started?
- Sign consent and give copy

**Background**

- Firstly, could you tell me about your experience with the use of PROMs?
- Have you (or the hospital you work in) collected PROMs before we begun this trial?
- YES
  - - What measures do you collect?
    - How do you use this data (dissemination: reports, meetings)?
    - How do you think this information should be used?
  - No
    - Can you explain to me any QI initiative in which you involved the patient?
    - What is your experience of QI initiatives in the hospitals you work in?

**Attitudes**

- What are your views on the collection and use of PROMs?
- In particular, what are your opinions on the use of PROMs as a QI tool?
- How would you feel if this data was used :
  - As a clinical governance tool in the hospital(s) you work in?
  - To inform patient choice by publically reporting the data?
  - To inform purchasers decisions?
  - To link payment to results?
- How do you think PROMs should be used?
- Would you like to receive regular feedback reports?

**Methodological issues**

- Moving on, one of the things I am particularly interested in is the thought process when you read the report? Could you explain this to me?
- In particular, what factors do you think affected the results (patient, surgeon, hospital)?
  - Do you agree with the findings?
  - What are your views on patients reporting on these issues?
- Did you understand the feedback report?
- What information did you find useful?
- Was there anything you did not understand?
- How could we improve the report?
- Did you find the feedback clinically meaningful?
- Would you use these measures to detect a change in outcomes over time or across surgeons?

**Impact**

- In theory, we assume that providing surgeons with benchmarked feedback will promote changes in patient care. How do you think this happens in practice?
  - Could you describe any changes you made/would like to make based on these findings?
  - Do you think this feedback would stimulate further research/ audit?
  - Steps taken to implement changes?
  - If no, what factors may prevent change?
- How else could PROMs feedback impact on care or practice?
- There is a debate about the level at which PROMs should be fed back (surgeon/hospital), could you describe your opinion on this matter?

**Practical issues**

- From a practical point of view, is the routine collection of PROMs is feasible?
  - Administration, coding, analysis, interpretation?
- What would facilitate the collection and use of PROMs?
  - Support required to collect and appropriately use the information?
  - Guidelines or training/educational needs?
  - Role of technology?

Thanks for sharing your views and experience with me. Have you any additional questions or anything else to add before we finish?
